# An update on the management of axial spondyloarthritis for sports medicine professionals

**DOI:** 10.1186/s13102-024-00998-z

**Published:** 2024-10-07

**Authors:** Abhijeet Danve, Marina Magrey, Atul Deodhar

**Affiliations:** 1 Rheumatology, Yale School of Medicine, 300 Cedar Street, TACS-525, New Haven, CT 06520 USA; 2 University Hospitals Cleveland Medical Center, Case Western Reserve University School of Medicine, Cleveland, OH USA; 3 Oregon Health & Science University, Portland, OR USA

**Keywords:** Axial spondyloarthritis, NSAIDs, Sports medicine, Treatment

## Abstract

**Background:**

Axial spondyloarthritis (axSpA) is a chronic inflammatory disease which mainly affects the spine and sacroiliac joints, causing longstanding back pain, stiffness, and limited mobility. AxSpA is an underrecognized disease in non-rheumatology practices because of its heterogeneous clinical features that may be difficult to identify.

**Main body:**

Sports medicine practitioners are well positioned to suspect and recognize axSpA among their patients with chronic back pain and refer them to a rheumatologist. Early referral to a rheumatologist is important for timely diagnosis, prompt treatment, and improved long-term outcomes for patients with axSpA. Physical therapy and nonsteroidal anti-inflammatory drugs (NSAIDs) remain the first-line treatment for and the cornerstone of axSpA management. For patients with inadequate response to or intolerance of NSAIDs, biologic disease-modifying antirheumatic drugs (bDMARDs) and targeted synthetic (ts) DMARDs are indicated. These drugs can reduce pain, inflammation, fatigue, and disability and can improve health-related quality of life. The goal of this review is to improve awareness of axSpA among sports medicine practitioners and other non-rheumatologists so that these providers ensure timely referral of patients with suspected axSpA to rheumatologists for appropriate treatment and better outcomes. We also provide an update on current treatment possibilities for axSpA and describe how rheumatologists use treatment guidelines and disease activity measures to identify and optimally treat patients with active axSpA.

**Conclusion:**

Sports medicine practitioners have an excellent opportunity to identify patients with suspected axSpA and refer them to rheumatologists in a timely manner, as well as monitor symptoms among patients diagnosed with axSpA to identify inadequately controlled disease.

## Background

Sports medicine practitioners, general practitioners, first contact practitioners, osteopaths, physiotherapists and physical medicine and rehabilitation physicians frequently treat patients with low back pain, which is one of the most common musculoskeletal problems globally and is the fifth most common reason patients see a physician [1]. For the majority of these patients, low back pain is due to mechanical causes; however, the underlying cause for some patients may be axial spondyloarthritis (axSpA) [2, 3]. AxSpA is a chronic, immune-mediated inflammatory disease that primarily affects the axial skeleton and is characterized by chronic inflammatory back pain (IBP), stiffness, fatigue, reduced physical activity, and impaired quality of life [4, 5]. Uncontrolled inflammation in patients with axSpA can result in structural damage in the form of new bone formation and fusion of the sacroiliac joint (SIJ) and spine [6], but adequate disease control with cyclooxygenase-2 (COX-2) specific nonsteroidal anti-inflammatory drugs (NSAIDs) and biologics can reduce or inhibit structural damage, further emphasizing the importance of early referral to rheumatologists for timely diagnosis and prompt treatment.

Patients with axSpA typically experience chronic back pain that starts before the age of 45 years (most frequently between 20 and 30 years of age) [7]. Additionally, patients with axSpA may experience peripheral inflammatory arthritis, enthesitis, dactylitis, and extramusculoskeletal manifestations, including uveitis, psoriasis, and inflammatory bowel disease (IBD; Table 1) [8]. The prevalence of axSpA in the US population is estimated at 0.9–1.4% and 0.66–1.3% in the UK; diagnostic prevalence has been found to be only 0.2–0.7% [9, 10], suggesting vast underrecognition of axSpA in clinical practice [11]. AxSpA can be classified as either radiographic axSpA (also termed ankylosing spondylitis [AS]) or non-radiographic axSpA (nr-axSpA) based on the presence or absence of radiographic sacroiliitis as seen on plain x-rays of sacroiliac joints (modified New York Criteria: grade 2 bilateral, or grade 3 or 4 unilateral or bilateral) which include sclerosis, erosion, joint space narrowing, widening and fusion (radiographic sacroiliitis) [12]. Patients with nr-axSpA often experience longer diagnostic delays than patients with AS (7.1 years vs. 6.3 years) due to the absence of definitive radiographic findings [13]. The majority of patients with axSpA contact a healthcare provider within 1 year of symptom onset but still experience diagnostic delays that range from 5 to 14 years due to failure by physicians at the primary- and secondary-care levels to recognize the disease because of a lack of awareness of axSpA and its heterogeneous clinical features that may be difficult to identify [14].

**Table 1:** Typical musculoskeletal and nonmusculoskeletal features of AxSpA and clinical characteristics that indicate a referral is warranted

Typical Signs and Symptoms of AxSpA	
Musculoskeletal	Nonmusculoskeletal
IBP Neck pain Peripheral inflammatory arthritis Dactylitis Enthesitis (e.g., Achilles tendonitis or plantar fasciitis) Anterior chest wall pain	Uveitis Psoriasis (including nail lesions) Inflammatory bowel disease (Crohn’s disease and ulcerative colitis)
**Characteristics That Indicate a Referral Is Warranted**	
Chronic back pain with onset at ≤ 45 years of age Morning stiffness > 30 min Improvement with exercise HLA-B27 positive Peripheral or extramusculoskeletal manifestations Family history of spondyloarthritis Sacroiliitis	

AxSpA, axial spondyloarthritis; HLA-B27, human leukocyte antigen B27; IBP, inflammatory back pain

NSAIDs are typically recommended as first-line treatment for patients with axSpA, generally in combination with physical therapy and exercise [15–17], but > 40% of patients with axSpA have an inadequate response to or intolerance of NSAIDs [18]. For patients with active disease despite physical therapy and NSAIDs, several biologic and targeted synthetic disease-modifying antirheumatic drugs (b/tsDMARDs) have been approved by the US Food and Drug Administration, the European Medicines Agency, and the UK Medicines and Healthcare products Regulatory Agency [17]. Patients with axSpA generally have a better response to these therapies when treatment is initiated early in the disease process [17].

The purpose of this review is to improve awareness of axSpA among sports medicine practitioners and other non-rheumatologists caring for patients with chronic back pain so that they can identify patients with suspected axSpA and provide timely referral to rheumatologists for early initiation of treatment. We also provide an update on the current treatment landscape for axSpA and highlight how rheumatologists use current treatment guidelines and disease activity measures to identify and effectively treat patients with active axSpA.

## Main text

### Search strategy

To identify publications relevant to the management of axSpA, a series of PubMed searches were conducted covering articles published through November 2022. MeSH search terms included variations of axSpA (*axial spondyloarthritis*,* axSpA*,* ankylosing spondylitis*,* AS*,* non-radiographic axSpA*,* nr-axSpA*) in combination with other relevant variations of disease and treatment terms (e.g., *nonsteroidal anti-inflammatory drug*,* disease activity*,* response*,* therapy*,* treatment*,* biologic DMARD*,* Janus kinase*,* tumour necrosis factor*, *interleukin 17*) to focus the literature search on relevant publications. Publications mentioning disease activity measures, clinical features, disease progression, treatment guidelines, and treatment response were considered for inclusion; articles deemed irrelevant by the authors were excluded. Publications that were cited within relevant articles or otherwise identified by the authors that fit the criteria above were also included.

### Difficulties in diagnosing and evaluating AxSpA in the clinic

While an estimated 13–20% of the general population worldwide suffers from chronic lower back pain, only approximately 5% of patients with chronic back pain seen in primary care settings have axSpA [9, 19]. For the majority of patients with chronic back pain seen by clinicians, their symptoms are due to various mechanical causes (such as degenerative arthritis, disc disease, spinal stenosis, or postural back pain) rather than IBP (which is present in approximately 75% of patients with axSpA) [14]. Most clinicians have trouble differentiating between these two types of back pain due to a lack of awareness of the differences between mechanical and inflammatory patterns of back pain [20]. Non-rheumatologists are the first healthcare providers seen by approximately 35% of patients experiencing IBP symptoms [3, 21], but these providers often do not refer such patients to rheumatologists for further evaluation and instead prescribe NSAIDs and recommend exercise for low back pain [22, 23].

IBP is characterized by chronic (> 3 months’ duration) back pain starting before 45 years of age, insidious onset, worse pain at night, improvement with physical activity but not with rest, a good response to NSAIDs (> 50% relief in 48 h with full dose), morning stiffness for > 30 min, and presence of alternating buttock pain [24]. Two important caveats apply: all of these clinical features may not be present in a patient with IBP, and even more importantly, the presence of IBP alone does not mean that the person has axSpA. Only a small minority of patients with IBP (approximately 15%) have axSpA, as was shown by a population-based study in the US [25]. However, the presence of IBP should raise suspicion and indicate that the physician needs to evaluate the possibility of axSpA by comprehensive patient history. For patients with IBP, physicians should elicit patient and family history of extramusculoskeletal manifestations associated with axSpA, such as anterior uveitis, psoriasis, or IBD, since the presence of these features and IBP increases the likelihood of axSpA.

It is important for sports medicine practitioners and other non-rheumatologists to understand their local rheumatology referral procedures in order to follow the proper processes when referring patients with suspected axSpA to a rheumatologist [26]. Physical examination has a limited role in confirming inflammation in the SIJ or spine. Clinicians often rely on manual tests such as pain provocation, spinal mobility, and functional tests, but none of these reliably differentiate between mechanical and inflammatory back pain [9]. Clinicians should also look for tender or swollen joints, tender Achilles tendon or plantar fascia insertion, dactylitis, or psoriatic skin or nail lesions in their patients with suspected axSpA (Table 1) [24]. Although human leukocyte antigen B27 (HLA-B27) positivity or elevated C-reactive protein (CRP) serum levels increase the likelihood of axSpA, these tests alone are not confirmatory. Approximately 20% of patients with AS and as high as 50% of patients with nr-axSpA are HLA-B27 negative, and 50% of all patients with axSpA will have normal CRP levels [24]. For patients with clinical features suggestive of axSpA, a sports medicine practitioner should order a single anterior to posterior pelvis x-ray to look for radiographic sacroiliitis before referring a patient to a rheumatologist. When x-ray of the SIJ is normal or inconclusive but there is a high suspicion of axSpA, rheumatologists often order magnetic resonance imaging (MRI) of the SIJ. Clinicians should refer patients with chronic back pain that had an insidious onset and started before the age of 45 years to rheumatologists if these patients have one of the following features: HLA-B27 positivity, current IBP, peripheral or extramusculoskeletal manifestations, family history of spondyloarthritis, elevated markers of inflammation, or imaging (x-ray or MRI) evidence of sacroiliitis [9, 27, 28].

AxSpA has historically been underrecognized in women, most likely due to the belief that AS is a “man’s disease” [29]. The clinical presentation also varies between men and women. Women with axSpA more frequently present with enthesitis; have additional neck, pelvic, heel, or widespread pain; and tend to have more IBD, psoriasis, and dactylitis than men [29]. Women tend to have less radiographic progression than men and hence mostly present in the non-radiographic stage of the disease. In sports medicine practice, it is important to keep axSpA in the differential diagnoses for a female patient presenting with recurrent soft-tissue pain symptoms such as Achilles tendonitis or plantar fasciitis with no apparent cause—any of which may in fact be undiagnosed enthesitis. Sometimes patients may not even complain of back or neck pain unless specifically asked; even then, back pain may be dismissed as being due to a sports injury or “excessive training”. In contrast, men with axSpA are more likely than women to present with IBP as the first symptom, radiographic sacroiliitis typical of AS, and spinal radiographic changes such as syndesmophytes [29].

### Treatment guidelines for AxSpA

**Fig. 1:**
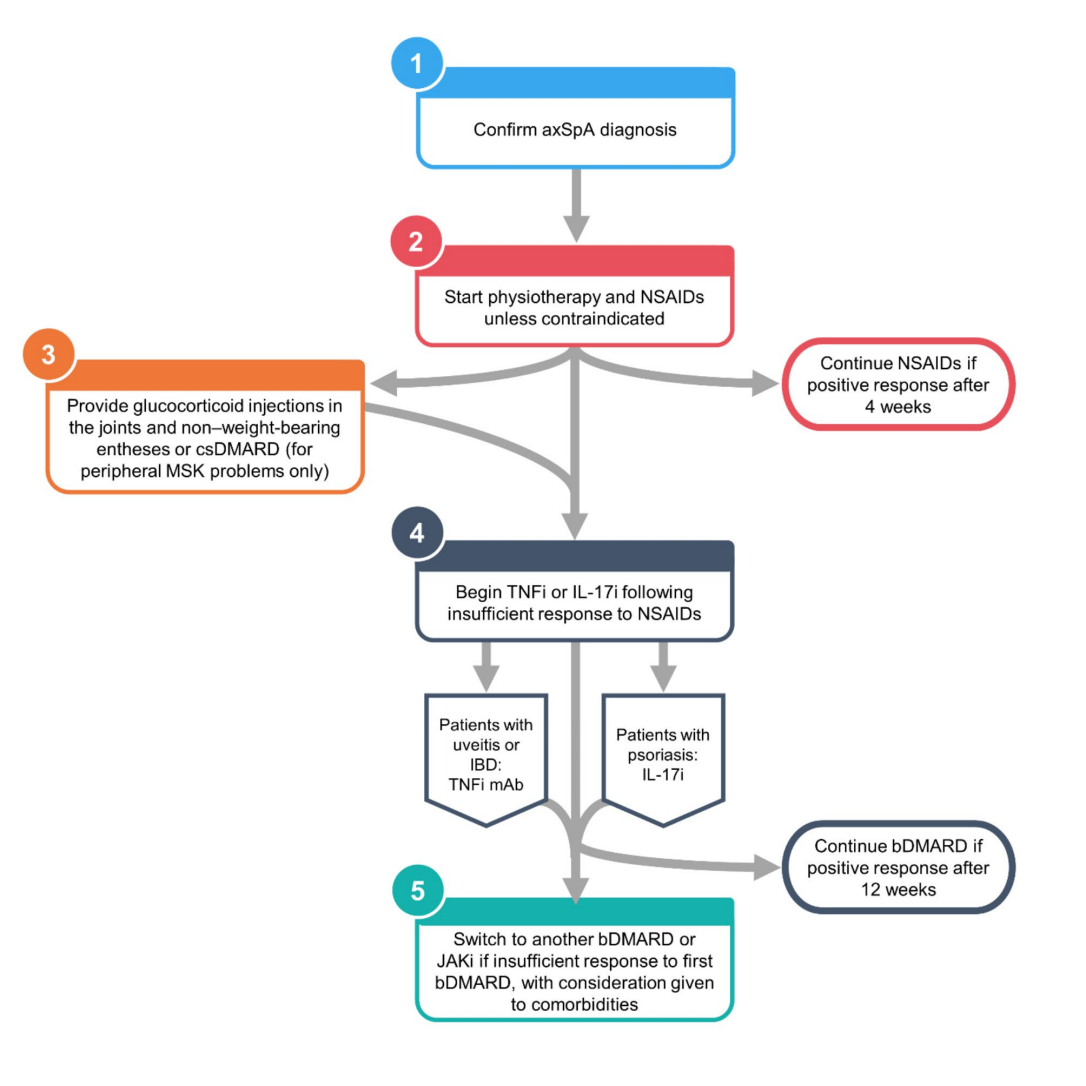
Decision tree for prescribing treatment for patients with axSpA AxSpA, axial spondyloarthritis; bDMARD, biologic disease-modifying antirheumatic drug; csDMARD, conventional synthetic disease-modifying antirheumatic drug; IBD, inflammatory bowel disease; IL-17i, interleukin 17 inhibitor; JAKi, Janus kinase inhibitor; mAb, monoclonal antibody; MSK, musculoskeletal; NSAID, nonsteroidal anti-inflammatory drug; TNFi, tumour necrosis factor inhibitor

These guidelines are provided here so that sports medicine practitioners and other non-rheumatologists are aware of the escalating treatment options that rheumatologists use to control signs and symptoms of axSpA (Fig. 1). Timely referral of patients with suspected axSpA to rheumatology by non-rheumatologists ensures earlier axSpA diagnosis and initiation of appropriate treatment by a rheumatologist, which improves the likelihood of good treatment outcomes.

In 2019, the American College of Rheumatology (ACR), the Spondylitis Association of America (SAA), and the Spondyloarthritis Research and Treatment Network (SPARTAN) provided updated treatment recommendations for patients with axSpA [16]. These recommendations were published prior to the approval of ixekizumab, tofacitinib, upadacitinib, and bimekizumab for AS and before all approvals for nr-axSpA in the US or EU. In 2022, the Assessment of SpondyloArthritis international Society (ASAS) and the European Alliance of Associations for Rheumatology (EULAR) also released updated guidelines for the treatment of axSpA [15].

Both ACR/SAA/SPARTAN and ASAS/EULAR guidelines recommend that regular physical exercise should be encouraged and referral to physiotherapy made for patients with axSpA [15, 16]. Despite being mostly unaware of these recommendations, patients generally understand the importance and benefits of physical activity as a foundation of their treatment regimen [30]. Supervised physiotherapy and exercise can improve functional capacity, reduce disease activity, reduce symptom severity, and improve quality of life [31, 32]. Patients who participate in supervised group physiotherapy have improved mobility and physical function and higher adherence rates than patients who perform individual, unsupervised exercise. However, patients who follow either exercise program have improved disease outcomes compared with patients who do not exercise (independent of pharmacological treatment) [33–35]. Exercise programs that utilize cardiorespiratory and strength training have shown positive benefits in objective signs of inflammation, joint damage, and symptoms in patients with inflammatory rheumatic diseases [36]. Additionally, disease education and lifestyle modifications such as smoking cessation remain a cornerstone of treatment as recommended by the ASAS-EULAR guidelines.

NSAIDs are recommended as first-line pharmacologic treatment for patients with active disease. Patients with active axSpA should receive continuous NSAID treatment, with no preference given between the different NSAIDs. Despite their status as first-line treatment for axSpA, NSAIDs have several potential safety issues that should be considered for patients with comorbidities such as IBD or chronic kidney disease, those with risk factors for gastrointestinal or renal complications, or those who are taking certain medications [37–41]. Although the risk of cardiovascular complications from NSAIDs for patients with axSpA remains a concern [42, 43], some evidence suggests that NSAID use may be inversely correlated with the risk of cardiovascular mortality for patients with axSpA [44, 45].

Glucocorticoid injections in the joints (including the SIJ) or non–weight-bearing entheses may be considered but long-term treatment with systemic glucocorticoids should be avoided [15, 16]. Even though glucocorticoid injections have not been tested on arthritis or enthesitis in patients with axSpA, ASAS-EULAR task force members are of the opinion that they can be efficacious. Conventional synthetic DMARDs such as methotrexate, sulfasalazine, and hydroxychloroquine should be avoided for patients with purely axial disease since they have failed to demonstrate efficacy in these patients. Sulfasalazine is conditionally preferred over methotrexate and may be prescribed for patients with predominantly peripheral disease [46].

Published treatment guidelines recommend bDMARDs (specifically tumour necrosis factor [TNF] and interleukin [IL]–17 inhibitors) and Janus kinase (JAK) inhibitors as second-line therapies for patients with axSpA who have an inadequate response to NSAIDs. These second-line treatments include TNF inhibitors such as adalimumab, certolizumab pegol, etanercept, golimumab, and infliximab; IL-17 inhibitors such as ixekizumab, bimekizumab, and secukinumab; and the JAK inhibitors tofacitinib and upadacitinib (Table 2) [17].

**Table 2:** Approved pharmacological treatment options for axSpA

Treatment	Administration	EMA approval		FDA approval	
		AS	nr-axSpA	AS	nr-axSpA
**TNF inhibitor** ^**a**^					
Adalimumab	40 mg SC q2w	✓	✓	✓	–
Certolizumab pegol	200 mg SC q2w or 400 mg SC q4w	✓	✓	✓	✓
Etanercept	50 mg SC qw	✓	✓	✓	–
Golimumab	50 mg SC q4w; 2 mg/kg IV at weeks 0 and 4, then q8w thereafter	✓	✓	✓	–
Infliximab	5 mg/kg IV at weeks 0, 2, and 6, then q6w thereafter	✓	–	✓	–
**IL-17 inhibitor** ^**b**^					
Ixekizumab	*AS*: 160 mg SC at week 0, then 80 mg q4w thereafter *nr-axSpA*: 80 mg SC q4w	✓	✓	✓	✓
Secukinumab	*With loading dose*: 150 mg SC at weeks 0, 1, 2, 3, and 4, then q4w thereafter *Without loading dose*: 150 mg SC q4w^c^	✓	✓	✓	✓
Bimekizumab	160 mg SC q4w	✓	✓	–	–
**JAK inhibitor**					
Tofacitinib	5 mg oral bid	✓	–	✓	–
Upadacitinib	15 mg oral qd	✓	✓	✓	✓

^a^ TNFi inhibitor monoclonal antibodies are recommended over other bDMARDs for patients with a history of uveitis or active IBD

^b^ IL-17 inhibitors may be preferred to other bDMARDs for patients with significant psoriasis but are not recommended in patients with active IBD

^c^ For patients with active AS despite treatment with secukinumab 150 mg, increasing the dose to 300 mg may be considered

AS, ankylosing spondylitis; axSpA, axial spondyloarthritis; bid, twice a day; EMA, European Medicines Agency; FDA, US Food and Drug Administration; IL-17, interleukin 17; IV, intravenously; nr-axSpA, non-radiographic axial spondyloarthritis; q2w, every 2 weeks; q4w, every 4 weeks; q6w, every 6 weeks; q8w, every 8 weeks; qd, once daily; qw, once weekly; SC, subcutaneously; TNF, tumour necrosis factor

ACR/SAA/SPARTAN treatment guidelines give no preference between different TNF inhibitors, except that TNF inhibitor monoclonal antibodies are recommended over other bDMARDs for patients with recurrent uveitis or IBD [16]. IL-17 inhibitors could be considered alternative bDMARDs if TNF inhibitors are contraindicated. For patients with secondary nonresponse to a TNF inhibitor (i.e., efficacy lost over time after initial response), switching to another TNF inhibitor is recommended. For patients who have primary nonresponse to a TNF inhibitor (i.e., no response to treatment), treatment with an IL-17 inhibitor can be considered. For patients with active axSpA and ulcerative colitis or Crohn’s disease and for whom a TNF inhibitor is contraindicated, tofacitinib is recommended over IL-17 inhibitors.

The more recent ASAS/EULAR guidelines recommend that TNF, IL-17, or JAK inhibitors should be considered as second-line therapy for patients with an inadequate response to NSAIDs and consistently high disease activity, with current practice being to start a TNF or IL-17 inhibitor first [15]. For patients with a history of uveitis or active IBD, TNF inhibitor monoclonal antibodies are recommended over other bDMARDs; IL-17 inhibitors are not recommended in patients with active IBD. IL-17 inhibitors may be preferred to other bDMARDs for patients with significant psoriasis due to the superiority of IL-17 inhibitors over TNF inhibitors in treating psoriasis. Patients should be continued on the bDMARD or JAK inhibitor if they have a clinically important improvement in (ASDAS; see following section) after ≥ 12 weeks and a positive rheumatologist’s opinion; otherwise, switching to another bDMARD or JAK inhibitor should be considered, with consideration given to the presence of comorbidities.

### Determination of disease activity in AxSpA

The overarching principle for effective treatment of axSpA is improving health-related quality of life by alleviating symptoms and inflammation, improving function, decreasing complications, maintaining normal work and daily function, and preventing structural damage [15, 16]. By understanding how rheumatologists determine disease activity (Table 3), sports medicine practitioners can help identify patients who need therapy escalation (Table 4).

**Table 3:** Disease activity and patient-reported outcome measures commonly used by rheumatologists for patients with axSpA

Disease Activity Measure	Assessment	Format	Interpretation
Single-item questionnaires	• PGA • Spinal pain	VAS or NRS (scale of 0–10)	• < 50% improvement in pain after treatment indicates active disease • PGA or pain score > 4 indicates active disease
BASDAI	Back pain, fatigue, peripheral joint pain and swelling, localized tenderness, severity and duration of morning stiffness scored using a 10-cm VAS	The BASDAI score (scale of 0–10) is the overall mean of individual scores from a 6-item questionnaire	• A score of ≥ 4 indicates active disease • Other common endpoints: ≥ 50% improvement from baseline (BASDAI50); achievement of MCID
ASDAS	Back pain, duration of morning stiffness, PGA, peripheral joint pain and swelling, and CRP (or rarely ESR)	ASDAS is a composite score (scale of 0–10) calculated from PROs and CRP measurement	• A score of < 1.3 indicates inactive disease • A change of ≥ 1.1 from baseline is considered a clinically important improvement • Other common endpoints: low disease, < 2.1; high disease, ≥ 2.1 to ≤ 3.5; very high disease, > 3.5
RAPID3	Pain, functional impairment, and PGA	The RAPID3 score (scale of 0–10) is calculated as the weighted sum of the individual PROs	• A score of ≥ 3.33 indicates active disease

ASDAS, Ankylosing Spondylitis Disease Activity Score; axSpA, axial spondyloarthritis; BASDAI, Bath Ankylosing Spondylitis Disease Activity Index; CRP, C-reactive protein; ESR, erythrocyte sedimentation rate; MCID, minimum clinically important difference; NRS, numeric rating scale; PGA, patient global assessment; RAPID3, Routine Assessment of Patient Index Data 3; VAS, visual analog scale

**Table 4:** Indications of insufficient control of axSpA by NSAIDs

**Clinical**
High disease activity as measured by PGA, BASDAI, or ASDAS Persistent spinal pain No improvement in peripheral arthritis, enthesitis, or dactylitis Suspected hip involvement
**Laboratory**
Elevated CRP

ASDAS, Ankylosing Spondylitis Disease Activity Score; axSpA, axial spondyloarthritis; BASDAI, Bath Ankylosing Spondylitis Disease Activity Index; CRP, C-reactive protein; NSAID, nonsteroidal anti-inflammatory drug; PGA, patient global assessment

Patient-reported outcome (PRO) measures can help determine which patients have an insufficient response to NSAID treatment [47, 48]. Single-item questionnaires using a visual analog scale (VAS) or numeric rating scale (NRS) can be used serially to assess pain, fatigue, and patient global assessment (PGA) of the disease. Patients who are prescribed NSAIDs should show ≥ 50% improvement in pain or a pain or PGA score of < 4 out of 10.

The Bath Ankylosing Spondylitis Disease Activity Index (BASDAI) is a 6-item questionnaire that measures patient-reported levels of back pain, fatigue, peripheral joint pain and swelling, localized tenderness, and the duration and severity of morning stiffness [47]. Each question is scored on a 10-cm horizontal VAS, the overall mean of which provides the BASDAI score (range, 0–10). A cutoff score of ≥ 4 has been used to indicate active disease and has been a criterion for enrolment in randomised controlled trials of TNF inhibitors [49, 50], while a score < 4 indicates low disease activity.

ASDAS measures disease activity based on a composite score of domains that include patient-reported assessments of back pain, duration of morning stiffness, peripheral joint pain and/or swelling, and general well-being, as well as CRP or rarely erythrocyte sedimentation rate (ESR). Four ASDAS categories are used to define disease activity states: inactive (< 1.3), low (≥ 1.3 to < 2.1), high (≥ 2.1 to ≤ 3.5) and very high (> 3.5) [51]. An ASDAS score of ≥ 2.1 (high disease activity) despite treatment with NSAIDs indicates that bDMARD or JAK inhibitor treatment should be considered.

Routine Assessment of Patient Index Data 3 (RAPID3) is an assessment tool commonly used for patients with rheumatoid arthritis and psoriatic arthritis that incorporates patient-reported scores for pain, functional impairment, and PGA [52, 53]. RAPID3 is commonly used by many rheumatology practices for all patient visits and correlates well with other measures of axSpA disease activity including BASDAI and ASDAS, showing promise as an alternative and more rapid measure for assessing axSpA disease activity in everyday practice [54, 55]. A RAPID3 score of 3.33 corresponded to a BASDAI score of 4 [54].

Each of these measures has important limitations. BASDAI and RAPID3 are purely patient reported and do not use objective measurements. ASDAS relies on the measurement of CRP in blood samples, which is only elevated in approximately 50% of patients with axSpA [56]. Concomitant fibromyalgia, which is prevalent in approximately 15–30% of patients with axSpA [57], can cause overestimation of disease activity. New semiobjective measures such as the Pain, Physical Function, Patient Global and Physician Global (P4) index are feasible in clinical practice but not yet validated in axSpA [58]. The development of composite measures that incorporate musculoskeletal symptoms, extramusculoskeletal manifestations, and acute phase reactants would allow for more complete assessment of disease activity [15, 59].

MRI of the SIJ (or rarely of the spine) may help evaluate patients with persistent axSpA symptoms despite therapy when findings of active inflammation would change disease management [16]. MRI is not recommended for detecting subclinical inflammation in patients with stable disease and is not typically performed to detect enthesitis in the spine or SIJ [16]. Monitoring disease progression by MRI or obtaining spinal radiographs on scheduled intervals is not recommended for routine evaluation due to uncertain value and high costs [16].

Up to 25% of patients with axSpA have hip involvement, and these patients typically have more severe disease activity and worse functional impairment [60, 61]. Hip involvement frequently appears within 4 years of disease onset, which typically occurs at a younger age in these patients [61]. bDMARD or JAK inhibitor treatment should be urgently initiated when hip involvement is suspected in patients with axSpA because these treatments may reduce the probability of further radiographic damage [60, 62, 63].

Several predictors for good clinical response to TNF inhibitors have been identified in patients with axSpA, including HLA-B27 positivity, elevated ESR and CRP, higher inflammatory activity on spine and/or sacroiliac joints MRI, and higher BASDAI scores [17, 64–69]. Other predictors of TNF inhibitor response not directly related to disease activity include male sex, BMI < 25 kg/m^2^, shorter disease duration, and younger age at treatment initiation [65–68, 70, 71]. Some factors that predict good clinical responses to TNF inhibitors might also apply to IL-17 inhibitors, including HLA-B27 positivity, elevated CRP, shorter disease duration, and younger age [72]; however, more research is needed.

The risk of radiographic progression for patients with axSpA could factor into the decision to initiate bDMARD treatment. However, due to the heterogeneous nature of radiographic progression in axSpA, it is unclear how much weight should be given to prevention of radiographic progression as a treatment target compared with control of clinical symptoms and inflammation [17, 73]. Depending on when in the disease course treatment is initiated, TNF and IL-17 inhibitors can reduce structural changes and limit radiographic progression [74, 75].

### Outstanding challenges

Sports medicine practitioners should be aware of physical limitations that might impact treatment of patients with axSpA. Patients with AS are at increased risk of osteoporosis and vertebral facture, although the risk may be lower in patients who are receiving NSAIDs [76–78]. Compared with other patients, those with AS also have impaired core stability and balance [79], which can be improved by performing balance and stability exercises [80].

Patients with a primary lack of response to treatment with a bDMARD are often continued on the same treatment for too long before being switched to another bDMARD or JAK inhibitor [81]. Due to this delay in switching treatments, these patients experience worse health-related quality of life, physical function, and work productivity than patients who responded to treatment [81]. Additionally, patients with nr-axSpA are treated less frequently with bDMARDs than patients with AS, despite having similar disease burden [82]. It is important for sports medicine practitioners and other non-rheumatologists to be familiar with indicators of inadequately controlled axSpA (chronic back pain that improves with exercise, morning stiffness, and peripheral or extramusculoskeletal manifestations) and clinical features of nr-axSpA in order to alert their patients’ rheumatologists.

## Conclusions

Sports medicine practitioners have an excellent opportunity to identify patients with suspected axSpA by improving recognition of the heterogeneous disease manifestations of axSpA. Patients with typical signs and symptoms of axSpA who should be referred include those with back pain with insidious onset before 45 years of age, morning stiffness, improvement with exercise, HLA-B27 positivity, peripheral or extramusculoskeletal manifestations, family history of spondyloarthritis, and sacroiliitis by x-ray or MRI if available. Timely referral of patients with suspected axSpA to a rheumatologist, as well as close symptom monitoring among patients already diagnosed with axSpA, will help these practitioners ensure their patients with axSpA are able to adequately control their disease. Knowledge of current disease activity measures along with clinical features of axSpA that indicate inadequate disease control can help these healthcare providers identify patients for whom NSAIDs and physical therapy are insufficient and refer them to a rheumatologist.

## Data Availability

No datasets were generated or analysed during the current study.

## Abbreviations

ACR: American College of Rheumatology
AS: Ankylosing spondylitis
ASAS: Assessment of SpondyloArthritis international Society
ASDAS: Ankylosing Spondylitis Disease Activity Score
axSpA: Axial spondyloarthritis
BASDAI: Bath Ankylosing Spondylitis Disease Activity Index
bDMARD: Biologic disease-modifying antirheumatic drug
CRP: C-reactive protein
ESR: Erythrocyte sedimentation rate
EULAR: European Alliance of Associations for Rheumatology
HLA-B27: Human leukocyte antigen B27
IBD: Inflammatory bowel disease
IBP: Inflammatory back pain
JAK: Janus kinase
MRI: Magnetic resonance imaging
nr-axSpA: Non-radiographic axial spondyloarthritis
NRS: Numeric rating scale
NSAID: Nonsteroidal anti-inflammatory drug
P4: Pain, Physical Function, Patient Global and Physician Global index
PGA: Patient global assessment
PRO: Patient-reported outcome
RAPID3: Routine Assessment of Patient Index Data 3
SAA: Spondylitis Association of America
SIJ: Sacroiliac joint
SPARTAN: Spondyloarthritis Research and Treatment Network
TNF: Tumor necrosis factor
tsDMARDs: Targeted synthetic disease-modifying antirheumatic drug
VAS: Visual analog scale
